# The lack of association between angiotensin‐converting enzyme gene insertion/deletion polymorphism and nicotine dependence in multiple sclerosis

**DOI:** 10.1002/brb3.600

**Published:** 2016-11-14

**Authors:** Sergej Nadalin, Alena Buretić‐Tomljanović, Polona Lavtar, Nada Starčević Čizmarević, Alenka Hodžić, Juraj Sepčić, Miljenko Kapović, Borut Peterlin, Smiljana Ristić

**Affiliations:** ^1^ Department of Biology and Medical GeneticsSchool of MedicineUniversity of RijekaRijekaCroatia; ^2^ Clinical Institute of Medical GeneticsUniversity Medical CentreLjubljanaSlovenia; ^3^ Postgraduate StudiesSchool of MedicineUniversity of RijekaRijekaCroatia

**Keywords:** angiotensin‐converting enzyme gene, multiple sclerosis, smoking

## Abstract

**Objective:**

Blood‐borne angiotensin II is generated from angiotensinogen via cleavage by renin and angiotensin‐converting enzyme (ACE), an enzymatic cascade known as the renin–angiotensin system (RAS). Several lines of evidence indicate that ACE, beyond its classical role of mediating blood pressure regulation, might contribute to the etiology of substance addictions by influencing dopaminergic signaling. A functional insertion/deletion (I/D) polymorphism of the ACE gene was associated with risk for being a smoker among individuals with depression and with smoking severity in studies comprising patients with depression and healthy controls. Several reports have described significantly increased ACE activity in cerebrospinal fluid and serum among MS patients. Furthermore, in our previous work with MS patients from Croatian and Slovenian populations, we demonstrated that the ACE‐I/D polymorphism contributes to an elevated MS risk among male patients. Here we investigated whether the ACE‐I/D polymorphism might influence smoking behavior among patients with MS.

**Patients and Methods:**

Genotyping was performed in 521 patients (males/females: 139/382) using polymerase chain reaction.

**Results:**

We revealed no significant differences in ACE genotype and allele frequencies between smokers and nonsmokers and no significant association between the ACE‐I/D polymorphism and either pack‐year smoking history or number of cigarettes smoked daily (*p *>* *.05, respectively).

**Conclusion:**

The ACE‐I/D polymorphism does not contribute either to risk for nicotine dependence or to smoking severity among MS patients. In the context of reports on the ACE‐I/D polymorphism and nicotine dependence among healthy controls and patients with depression, we may speculate that the mechanism by which this polymorphism influences nicotine dependence risk differs in MS compared to depression, although not compared to a healthy population. In addition to angiotensin II, other potential ACE substrates, such as substance P and neurotensin, which also influence dopaminergic neurotransmission (and are proposed to be associated with MS), may deserve study in future.

## 1 Introduction

Multiple sclerosis (MS) is a chronic autoimmune disease of the central nervous system in which myelin auto‐reactive targeting T‐lymphocytes drive an inflammatory process, leading to destruction of the myelin sheath, axonal loss, and neuronal degeneration (Ferreira et al., 2014; Mandia et al., 2014). Like other autoimmune disorders, MS is considered to be triggered by environmental factors in individuals with a genetic predisposition, and smoking has been considered one of the most established environmental risk factors for this illness (Mandia et al., 2014; Ramanujam et al., 2015). The relative risk of developing MS among smokers is almost twice that of never‐smokers, and patients with MS who smoke exhibit a more severe disease course and a faster disability progression rate (Correale & Farez, 2015; Fragoso, 2014; Healy et al., 2009; Manouchehrinia et al., 2013; Zhang et al., 2016).

Blood‐borne angiotensin II is generated from angiotensinogen via cleavage by renin and angiotensin‐converting enzyme (ACE), an enzymatic cascade known as the renin–angiotensin system (RAS) (Nawaz & Hasnain, 2008; Wosik et al., 2007). Several lines of evidence suggest that RAS, beyond its classical function in mediating the regulation of blood pressure, might act as an important determinant in the etiology of nicotine dependence by influencing dopaminergic signaling (Jenkins, Mendelsohn, & Chai, 1997; Jenkins et al., 1996; Obata, Takahashi, Kashiwagi, & Kubota, 2008). As a neurotransmitter, angiotensin II interacts with dopamine in mesocorticolimbic areas, and ACE modulates dopamine turnover in the rat striatum (Jenkins et al., 1996, 1997). In addition, decreased dopamine release in rat striatum has been reported in response to captopril and enalaprilat, antihypertensive drugs that inhibit ACE activity (Obata et al., 2008).

Current data regarding RAS‐related genes in the etiology of nicotine dependence are sparse (Baghai et al., 2008; Hubacek, Adamkova, Skodova, Lanska, & Poledne, 2004; Hubacek, Pitha, Skodova, & Poledne, 2001). A functional 287‐nucleotide fragment insertion/deletion (I/D) polymorphism in intron 16 of the ACE gene accounts for approximately 50% of ACE levels (Rigat et al., 1990), is the most studied RAS‐related polymorphic variant, and is the only RAS‐related polymorphism investigated in the etiology of nicotine dependence so far (Baghai et al., 2008; Hubacek et al., 2001, 2004). Although significantly greater risk for being a smoker has been observed among ACE‐DD homozygous individuals with depression in the German population (Baghai et al., 2008), studies comprising healthy subjects from the Czech Republic and German populations found no association between the ACE‐I/D polymorphism and smoking risk (Baghai et al., 2008; Hubacek et al., 2004). Furthermore, the ACE‐I/D polymorphism has been identified as possibly underlying smoking severity; the ACE‐DD homozygous genotype contributes to higher daily cigarette consumption among patients with depression as well as to greater pack‐years of smoking history among healthy individuals in the German population (Baghai et al., 2008).

To date, several reports have described significantly increased ACE activity in cerebrospinal fluid and serum among patients with MS (Constantinescu, Goodman, Grossman, Mannon, & Cohen, 1997; Kawajiri et al., 2008; Schweisfurth, Schiöberg‐Schiegnitz, Kuhn, & Parusel, 1987). Furthermore, in our previous work with MS patients from Croatian and Slovenian populations, we demonstrated that the homozygous ACE‐DD genotype might contribute to an elevated MS risk among male patients (Lovrečić et al., 2006). In line with these findings and according to the evidence suggesting the relevance of RAS in brain dopaminergic signaling (Jenkins et al., 1996, 1997; Obata et al., 2008), in the current work we aimed to investigate whether the ACE‐I/D polymorphic variant might influence smoking behavior among patients with MS. Although the detrimental effects of smoking in MS are well established (Ramanujam et al., 2015; Zhang et al., 2016) and genetics has been proposed to play a substantial role in vulnerability to smoking (Li, 2008), to the best of our knowledge, no studies elucidating the etiology of nicotine dependence in MS have been published.

## 2 Patients and methods

### 2.1 Study participants

Our study group consisted of 521 patients, all fulfilling Mc Donald's criteria for MS (Polman et al., 2005) and all recruited by collaborating genetic and clinical centers from Croatia and Slovenia, which belong to the same geographic area where the populations share a similar ethnic background (Table 1) (Zupan, Vrabec, & Glavač, 2013). The course of MS was classified according to the clinical data (Lublin & Reingold, 1996), and disease severity was estimated using the Expanded Disability Status Scale and Multiple Sclerosis Severity Score at the time when blood samples for genetics analysis were taken (Kurtzke, 1983; Roxburgh et al., 2005). Patients under treatment with ACE inhibitors were excluded from the study. Neurologists obtained information concerning the patients’ smoking status via a questionnaire. Smokers were defined as individuals who smoked more than one cigarette a day, and had smoked for more than one year. Nonsmokers were defined as those who had smoked less than 100 cigarettes during their lifetime (Guo et al., 2007; de Leon & Diaz, 2005). The numbers of quitters, occasional smokers, ex‐smokers and those who have been smoking for less than 1 year were too small for statistical analysis and were excluded from the study. To assess the severity of nicotine dependence, patients were also asked for pack‐year smoking history, which was calculated by number of cigarettes smoked per day × number of years smoked. The study was approved by the Ethics Committees of both genetic centers and conducted according to the ethical standards expressed in the latest version of the Declaration of Helsinki.

**Table 1:** Characteristics of patients with multiple sclerosis

	Males (*n *=* *139)	Females (*n *=* *382)
Age at onset	31.1 ± 8.7	31.4 ± 10.0
Age at blood sampling	44.1 ± 12.3	43.8 ± 12.0
Course (%)^a^		
Primary progressive	18 (12.9)	26 (6.8)
Secondary progressive	45 (32.4)	95 (24.9)
Relapsing–remitting	76 (54.7)	261 (68.3)
Expanded Disability Status Scale	4.0 ± 2.5	3.6 ± 2.2
Multiple Sclerosis Severity Score	4.5 ± 2.9	4.2 ± 2.8
Smoking behavior		
Smokers/nonsmokers	66/73	150/232
Pack‐year smoking history^b^	25.6 ± 17.3	14.2 ± 11.1
Number of cigarettes smoked per day^c^	21.0 ± 7.6	14.6 ± 7.6

Males vs. Females: ^a^χ2 = 9.6, *p *<* *.01; ^b^F = 25.3, *p *<* *.0001; ^c^F = 23.8, *p *<* *.0001.

### 2.2 Genotyping

Genomic DNA was extracted from whole blood using a FlexiGene DNA kit 250 (QIAGEN GmbH, Hilden, Germany) according to the manufacturer's instructions. Genotyping was performed by polymerase chain reaction (PCR) analysis using protocols previously described (Rigat et al., 1990). To exclude mistyping of the ACE‐ID heterozygotes as ACE‐DD homozygotes, all ACE‐DD genotype samples were additionally confirmed with insertion‐specific PCR (Shanmugam, Sell, & Saha, 1993).

### 2.3 Statistical analysis

The ACE genotype and allele distributions among smokers and nonsmokers, as well as observed and expected genotype proportions according to Hardy–Weinberg equilibrium, were compared by the chi‐square (χ*2*) test. The relationship between pack‐year smoking history and number of cigarettes smoked daily and ACE genotype was evaluated using a one‐way analysis of variance. Probability (*p*) values less than .05 (*p *<* *.05) were considered statistically significant. Statistical analyses were performed using Statistica for Windows, version 12.

## 3 Results

In line with gender‐specific differences in the effect of the ACE‐I/D polymorphism in various conditions and/or diseases, such as blood pressure (among healthy individuals) (Avila‐Vanzzini et al., 2015), hypertension (Higaki et al., 2000; Sipahi, Budak, Şen, Ay1, & Şener, 2006), schizophrenia (Mazaheri, 2015; Nadalin, Buretić‐Tomljanović, Ristić, Jonovska, & Tomljanović, 2015; Nadalin et al., 2012), as well as MS (Lovrečić et al., 2006), and according to observations of gender–gene interaction in risk for nicotine dependence in general population (Beuten, Payne, Ma, & Li, 2006; Beuten et al., 2005; Nedic et al., 2010; Tochigi et al., 2007) and specific diseases (i.e., schizophrenia) (Nadalin, Buretić‐Tomljanović, Rebić, Pleša, & Šendula Jengić, 2016) association analyses between ACE‐I/D polymorphism and smoking habits were performed separately among male and female patients. Furthermore, there is also evidence that estrogen may influence dopaminergic neurotransmission, since it has been observed that estrogen treatment reduces dopamine receptor D2 levels in several rat brain regions (Chavez et al., 2010).

A slightly greater prevalence of nicotine dependence observed among male patients did not reach statistical significance (*p *>* *.05, respectively); yet, data regarding smoking severity revealed significantly higher values of pack‐year smoking history and a significantly greater number of cigarettes smoked per day among male patients compared to females with MS (*p *<* *.0001 in both cases, respectively) (Table 1). The prevalence of nicotine dependence was elevated when compared to the general Croatian and Slovenian populations (Klemenc‐Ketiš & Kersnik, 2015; Loubeau, 2009; Turek et al., 2001). Table 2 shows the allele and genotype frequencies for the ACE‐I/D polymorphism according to patient smoking status. The statistical power of our study was 100% to detect a 1.5‐fold increase in the ACE‐D allele and ACE‐I allele frequency, respectively. The ACE genotype distributions in male or female patients, as well as in male smokers or nonsmokers, and female smokers or nonsmokers, were consistent with the Hardy–Weinberg equilibrium (*p *>* *.05, respectively). The ACE allele and genotype frequencies were similar to those reported in our previous study (Lovrečić et al., 2006) and did not deviate from those observed in the European population (http://www.ncbi.nlm.nih.gov/snp/?term=rs1799752). No significant differences were found in ACE genotype and allele distributions between total male and female patients (*p *>* *.05, respectively). ACE genotype and allele frequencies between smokers and nonsmokers also did not differ significantly in male or female patients (*p *>* *.05, respectively), and no significant differences in frequencies of ACE genotype and alleles were detected when the smokers (males vs. females) and nonsmokers (males vs. females) were analyzed separately. Finally, no significant association between the ACE‐I/D polymorphism and either pack‐year smoking history or number of cigarettes smoked daily was identified (*p *>* *.05, respectively) (Table 3).

**Table 2:** The frequency of ACE genotypes and alleles according to smoking status

		Genotypes (%)			Alleles (%)	
		DD	ID	II	D	I
Males	Smokers (*n *=* *66)	22 (33.3)	31 (47.0)	13 (19.7)	75 (56.8)	57 (43.2)
	Nonsmokers (*n *=* *73)	23 (31.5)	40 (54.8)	10 (13.7)	86 (58.9)	60 (41.1)
		χ^*2*^ = 1.2, *df* = 2, *p *=* *.55			χ^*2*^ = 0.1, *df* = 1, *p *=* *.73	
Females	Smokers (*n *=* *150)	44 (29.3)	78 (52.0)	28 (18.7)	166 (55.3)	134 (44.7)
	Nonsmokers (*n *=* *232)	61 (26.3)	127 (54.7)	44 (19.0)	249 (53.7)	215 (46.3)
		χ^*2*^ = 0.4, *df* = 2, *p *=* *.80			χ^*2*^ * *= 0.2, *df* = 1, *p *=* *.65	

ACE, angiotensin‐converting enzyme.

**Table 3:** The severity of nicotine dependence according to ACE‐I/D polymorphism

		ACE genotype			F	*p*
		DD	ID	II		
Pack‐year smoking history	Males	26.8 ± 17.0	27.4 ± 18.5	19.9 ± 15.2	0.7	.50
	Females	14.4 ± 9.3	14.0 ± 12.3	14.3 ± 10.2	0.0	.10
Number of cigarettes smoked per day	Males	21.6 ± 8.1	21.4 ± 6.4	19.2 ± 9.5	0.3	.71
	Females	14.2 ± 7.4	14.8 ± 7.9	14.9 ± 7.3	0.1	.91

ACE, angiotensin‐converting enzyme.

## 4 Discussion

Components of cigarette smoke have been associated with demyelination and axonal degeneration, and nicotine compromises the blood–brain barrier and exerts immunomodulatory action on T‐lymphocytes (Correale & Farez, 2015; Ramanujam et al., 2015; Zhang et al., 2016). In this study, which to our knowledge is the first to address the genetics of nicotine dependence among patients with MS, we investigated whether smoking behavior in MS might be influenced by a functional ACE‐I/D polymorphism that has proved to be relevant in both nicotine dependence and MS (Baghai et al., 2008; Lovrečić et al., 2006; Živković et al., 2016). In addition to our previous study that first identified the possible relevance of the ACE‐I/D polymorphism in MS, only a few reports on the role of the ACE‐I/D polymorphism in MS have been published so far (Hladikova, Vašků, Stourač, Benešová, & Bednařík, 2011; Klupka‐Sarić et al., 2011; Živković et al., 2016).

Since the homozygous ACE‐DD genotype is associated with tissue and plasma enzyme levels almost twice that of the homozygous ACE‐II genotype (Rigat et al., 1990), we initially speculated that individuals with the ACE‐DD genotype might release dopamine at higher levels and consequently could be more vulnerable to nicotine dependence. However, our results demonstrating no significant influence of the ACE‐I/D polymorphism on either smoking risk or the severity of nicotine dependence (Tables 2 and 3) do not argue in favor of our speculation. Our data are consistent with findings showing no association between the risk for nicotine dependence and the ACE‐I/D polymorphism among healthy individuals from the Czech Republic and German populations (Baghai et al., 2008; Hubacek et al., 2004), as well as with a negative report on the association between this polymorphism and number of cigarettes smoked per week among healthy controls from the Czech Republic (Hubacek et al., 2004). However, the results are in disagreement with previous findings from a German population study reporting a positive association of ACE‐DD homozygosity, nicotine dependence risk, and number of cigarettes smoked daily among patients with depression. They also are not in agreement with data showing a positive association between the ACE‐DD homozygous genotype and pack‐year smoking history among healthy individuals (Baghai et al., 2008). Thus, we may speculate that the plausible mechanism by which the ACE‐I/D polymorphic variant contributes to risk for developing nicotine dependence differs in MS when compared with depression, although not when compared with a healthy population (Baghai et al., 2008; Hubacek et al., 2004). On the other hand, although the plausible mechanism by which the ACE‐I/D polymorphism contributes to the severity of nicotine dependence may also differ in MS when compared to depression (Baghai et al., 2008), whether it differs in comparison to a healthy population is disputable because reports disagree about its influence on the number of cigarettes smoked per week and/or pack‐year smoking history among healthy subjects (Baghai et al., 2008; Hubacek et al., 2004). Finally, it is plausible that in addition to genetic factors, smoking habits among patients with MS, in comparison to healthy controls, may be modified by the illness itself; for instance, because of indisposition, patients with MS may smoke fewer cigarettes daily than before MS onset.

Advances in understanding of the RAS signaling pathway indicate an intriguing possibility that RAS, by modulating T‐lymphocytes mediated autoimmunity, may be implicated in the detrimental effects of smoking in MS (Correale & Farez, 2015). Several studies have observed that angiotensin II promotes inflammation and tissue injury, mediating key events in animal models of autoimmune disease (Marchesi, Paradis, & Schiffrin, 2008; Okunuki et al., 2009; Platten et al., 2009; Sagawa, Nagatani, Komagata, & Yamamoto, 2005; Stegbauer et al., 2009). Based on previous observations demonstrating that serum ACE activity increases significantly after smoking (Kitamura, 1987) and chronic smoking results in enhanced RAS activation in monozygotic twins, discordant for smoking (Laustiola, Lassila, & Nurmi, 1988), Correale and Farez (2015) (Correale & Farez, 2015) recently investigated whether RAS might be involved in immunomodulatory effects of smoking on MS progression. As expected, their findings argue in favor of smoking having modulator role in the expression and activity of RAS components: both auto‐reactive T‐lymphocytes and monocytes derived from patients who smoked produced significantly higher amounts of renin, ACE, as well as angiotensin II receptor type I (AT1R). Furthermore, they observed that inhibition of ACE using enalapril and blocking AT1R using antihypertensive losartan, down‐regulated interleukin (IL)‐17 and IL‐22 secreting cell numbers in MS patients who smoked. In smokers, losartan treatment was associated with improvement of MS prognosis, leading to a significant decrease in risk of conversion of clinically isolated syndrome to definite MS.

Several limitations of this study should be noted here. Our sample included a relatively small number of participants, thus leaving open the possibility that some minor effects were not detected. In addition, it consisted of an unbalanced number of male and female patients, which might have led to bias in the statistical analyses. Furthermore, the current sample also lacked a normal control group, and data regarding smoking behavior were based exclusively on patient self‐report. For confirmation of these findings, further studies are required that include other diseases and/or conditions, assess nicotine dependence with more specific methods, such as the Fagerstrom test, and investigate the relevance of other RAS‐related polymorphic variants in smoking behavior (e.g., those for angiotensinogen, angiotensin II receptors) (Hladikova et al., 2011; Živković et al., 2016). Moreover, because of a high prevalence of major depressive disorder reported among MS patients (Siegert & Abernethy, 2005) and in line with the positive association between smoking behavior and the ACE‐I/D polymorphism among patients with depression (Baghai et al., 2008), it would be interesting to investigate the relevance of the ACE‐I/D polymorphism among those patients separately from other MS patients. Another issue that should be considered is that ACE, in addition to angiotensin II, has other potential substrates, some of which influence dopaminergic neurotransmission (and are proposed to be associated with MS), such as substance P and neurotensin (Binder, Kinkead, Owens, & Nemeroff, 2001; Krasnova, Bychkov, Lioudyno, Zubareva, & Dambinova, 2000; Soltys, Knight, Scharf, Pitt, & Mao‐Draayer, 2014; Vilisaar et al., 2015).

In conclusion, we report negative findings regarding the influence of the ACE‐I/D polymorphism on smoking behavior among patients with MS. According to our results, this polymorphism does not contribute either to risk for developing nicotine dependence or to smoking severity in this patient group.
